# Associations between vitamin E, oxidative stress markers, total homocysteine levels, and physical activity or cognitive capacity in older adults

**DOI:** 10.1038/s41598-021-92076-4

**Published:** 2021-06-18

**Authors:** Ahmad H. Alghadir, Sami A. Gabr, Shahnawaz Anwer, Heng Li

**Affiliations:** 1 Rehabilitation Research Chair, College of Applied Medical Sciences, King Saud University, Riyadh, 11433 Saudi Arabia; 2 Department of Building and Real Estate, Faculty of Construction and Environment, Hong Kong Polytechnic University, Hung Hom, Kowloon, Hong Kong, SAR; 3 Depratment of Anatomy, Faculty of Medicine, Mansoura University, Mansoura, 35516 Egypt

**Keywords:** Biomarkers, Endocrinology

## Abstract

This study examined the associations between vitamin E, oxidative stress markers, total homocysteine levels, and physical activity or cognitive capacity in older adults. One hundred and six older adults (62 men, 44 women) within the age range of 56–81 years participated. The Global Physical Activity Questionnaire and the Loewenstein Occupational Therapy Cognitive Assessment were used to assess physical activity and cognitive function, respectively. Vitamin E (e.g., α-tocopherol and γ-tocopherol), oxidative stress markers (e.g., total antioxidant capacity and nitric oxide), and total homocysteine were estimated. There were significant associations between physical activity (high versus moderate versus poor) and all biomarkers (all *p* = 0.000, and *p* = 0.010 for γ-tocopherol). While total homocysteine and total antioxidant capacity were significantly associated with cognitive capacity (*p* = 0.000), vitamin E levels (e.g., α-tocopherol and γ-tocopherol) and nitric oxide (*p* = 0.354, 0.103 and 0.060, respectively) were not related to cognitive capacity in older adults. This study concludes that physical activity was associated with Vitamin E, oxidative stress markers, total homocysteine, and cognitive capacity in older adults. Although cognitive capacity was associated with total homocysteine and total antioxidant capacity, it was unrelated to vitamin E levels and nitric oxide in older adults.

## Introduction

Cognitive aging is one of the most common health care issues in older adults^1,2^. Every three seconds, a new case of dementia is detected worldwide^3^, and the number of individuals affected is projected to increase up to 132 million by 2050^3^. Aging is a diverse and multifaceted process that involves the biological, psychological and social components of human life^4^. According to the International Association of Gerontology and Geriatrics (IAGG) Consensus Report, predementia and Alzheimer's disease can hasten aging and are the leading causes of disability in older persons^5^. As a result, identifying accurate predictors of cognitive aging will aid in the development of an effective approach to reduce the negative impacts of cognitive aging and increase quality of life during the aging process.

The physiological manifestation of aging can present as a reduced function of the central nervous system and release of some biomarkers in the blood^6^. A growing body of evidence suggests that oxidative mechanisms, low vitamin E levels, and high total homocysteine (tHcy) levels play a role in the pathophysiology of cognitive aging^7–10^. While many theories support aging-related lower redox state and increased oxidative stress, the specific pathophysiology of aging remains unknown^8,11^. Oxidative stress enhanced by free radicals are important factors for the onset and progression of cognitive disorders including Alzheimer’s disease and mild cognitive impairment^12,13^. Vitamin E is the most important antioxidant which plays a vital role to protect the central nervous system against free radical-led damage^14^. Additionally, it has been suggested that oxidative stress is one of the factors causing aging^15^. Furthermore, oxidative stress may play a vital role in the pathophysiology of Parkinson’s disease, cancer, atherosclerosis, and Alzheimer disease^16^. According to a recent study, lower overall antioxidant capacity in serum resulted in decreased antioxidant capacity of the aged brain^9^. They also discovered a link between low blood total antioxidant capacity (TAC) and worse cognitive capability, elevated tHcy, and low physical activity levels^9^. According to many researches, the most prevalent characteristics that can speed the progression of aging include impaired cognitive function, increasing tHcy levels, and a lack of physical exercise^17,18^. A recent study also found that a high tHcy level is a definite and modifiable risk factor for cognitive impairment and dementia^19^. Furthermore, homocysteine can interact with both risk and protective factors, enabling for the identification of high-risk individuals and the development of early intervention strategies^20^. Importantly, in older persons, the tHcy level in plasma is the most reliable biomarker of cognitive function and tissue degradation^21^.

Reduced cognition in the elderly is associated with decreased independence and a negative quality of life^22^. Previous research has shown that physical activity (PA) enhances neurocognitive function and reduces the risk of neurodegenerative illnesses in older persons^23,24^. Additionally, PA has been identified as one of the most effective lifestyle activities for lowering the risk of dementia and increasing cognitive function^25^. Furthermore, past research has shown that PA intervention improves neurocognitive performance in older persons^26,27^. Moreover, PA levels earlier in life have been linked to cognitive function, dementia risk, and mild cognitive impairment later in life^28,29^. Previous randomized controlled trials found that exercise improves cognitive function. Heyn et al.^30^ published a meta-analysis of 30 randomized controlled trials and discovered that exercise has a significant favorable effect on cognitive function. Similarly, a prior review found that PA could help prevent age-related cognitive impairment and neurodegenerative illnesses^31^. While some studies have discovered a relationship between PA levels and cognitive capability, there is limited evidence that PA levels alter biomarkers or cognitive performance in older persons. Two recent Cochrane reviews, for example, found very little data on the effects of vitamin and mineral supplements in older persons with mild cognitive impairment^32,33^. Because cognitive abilities are important for maintaining a high quality of life as we aged^34^, this study examined the relationships between vitamin E, oxidative stress markers, total homocysteine levels and physical activity or cognitive capacity in older persons.

## Materials and methods

### Participants

**Figure 1:**
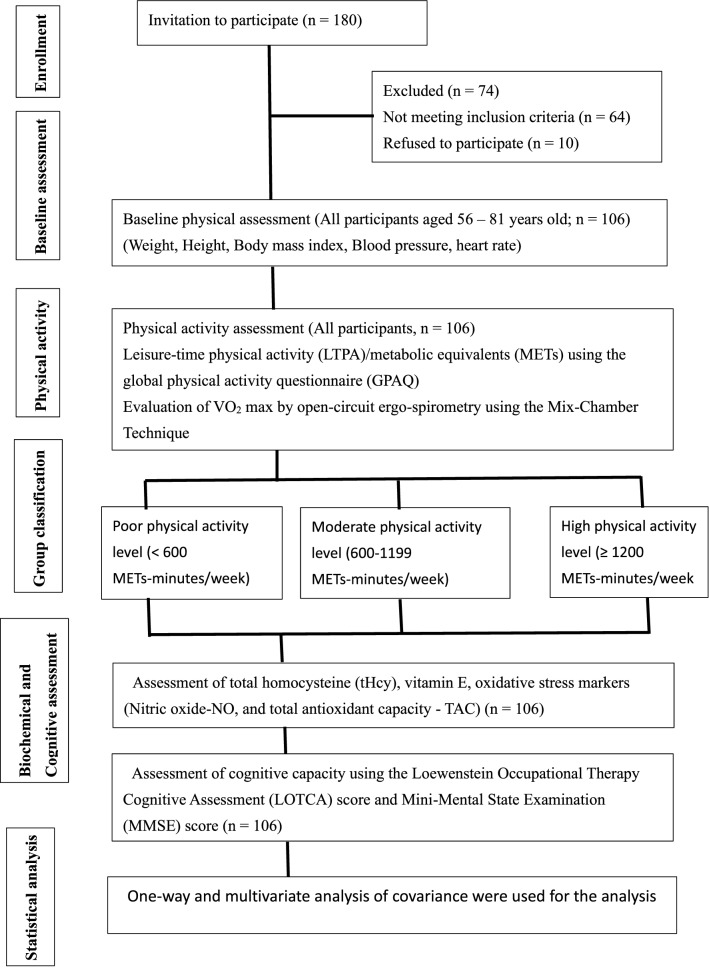
Outline of study procedures.

One hundred and eighty older adults were invited from the community using a convenient sampling method. Only 106 participants (62 men, 44 women) within the age range of 56–81 years were eligible to participate as per the inclusion criteria. Participants were excluded if they had a physical inability; physiological, psychological, immunological, and metabolic diseases; severe dementia [Mini-Mental State Examination (MMSE) score ≤ 10]^35^; or were using glucocorticoid medication. The participants were divided into three groups based on their PA scores: inactive or sedentary ($n = 29$), moderately active ($n = 37$), and highly active ($n = 40$). Furthermore, the current study's participants were separated into three groups (i.e., good, moderate, and poor) based on their cognitive capacity as determined by the Loewenstein Occupational Therapy Cognitive Assessment (LOTCA) scores. No one in the study was using hormone therapy, such as replacement therapy or treatment for male pattern baldness. Figure 1 depicts an outline of the research procedure. The ethical committee of the Rehabilitation Research Chair, King Saud University, Riyadh, Saudi Arabia (approval number: RRC-2014-016) approved this study. All experiments were carried out in line with the Helsinki Declaration. An informed consent form was completed and signed by all participants.

### Assessment of physical activity

The Global Physical Activity Questionnaire (GPAQ) was administered by trained research personnel to assess the PA levels. The GPAQ consists of 16 items designed to assess the amount of PA among three domains including work, transportation, and leisure time activities during a typical active week. The GPAQ was developed by WHO to determine the total amount of moderate and vigorous intensive activity in a normal week^36^. The participant’s leisure-time physical activity (LTPA) was measured using metabolic equivalents (METs), as previously reported^37,38^. A MET is defined as “the amount of oxygen consumed while sitting at rest” and is approximately equal to 3.5 ml oxygen/kg/minute^39^. The frequency and duration of LTPA (minutes/day) participation over a typical week was recorded. The GPAQ data was cleaned and processed in accordance with the GPAQ analysis guidelines^36^. According to WHO recommendations, the participants were classified into three groups depending on their total PA levels per week^36^: (1) Physically inactive (< 600 METs-minutes/week), (2) Moderately active (600–1199 METs-minutes/week), and (3) Highly active (≥ 1200 METs-minutes/week).

### Assessment of maximum aerobic capacity (VO_2_ max)

The maximum aerobic capacity (VO_2_ max) was also assessed in order to confirm the PA levels obtained by LTPA utilizing the GPAQ. The VO_2_ max was defined as the highest amount of oxygen consumed in a particular unit of time when exerting maximum effort (e.g., ml O_2_/kg body weight/minute)^40^. Prior to VO_2_ max testing, participants' baseline cardiovascular health, exercise capacity, and response to exercise were determined by assessing blood pressure using the published protocols^41,42^. Subsequently, the participants completed a 3-min warmup activity on a treadmill at 3.1 km/h. The VO_2_ max was determined using an open-circuit ergo-spirometry device, as described in the literature^43,44^. Following the warmup, the participants engaged in active exercise on a treadmill (inclination 1%), with a starting velocity of 4.5 km/h gradually increasing at 0.5 km/minute. The active exercise was either continued until exhaustion or ended when the individuals met the following criteria: (1) an increase in VO_2_ max remained 2 ml/kg even after increasing exercise intensity (i.e., they reached a plateau stage); (2) expiratory exchange ratio 1.1; or (3) participants reached their maximum heart rate (220-age), as measured in previous studies^43,44^. Ergo-spirometry was used to determine the VO_2_ max during the physical exercise. Afterward, all participants completed a 10-min cool-down activity in which the treadmill velocity was gradually reduced until the participant's blood pressure and heart rate recovered to normal resting levels. A portable heart rate monitor (Polar Electro, Kempele, Finland) was used to continually measure heart rate, which aids in keeping exercise intensity within the range of the predicted training heart rate^45^. All tests were carried out by trained research staff.

### Assessment of cognitive capacity

Trained research personnel assessed the cognitive capacity of the participants using the Loewenstein Occupational Therapy Cognitive Assessment (LOTCA) score and MMSE score. The pre-validated MMSE was used to measure cognitive capacity among older populations, as previously reported^46,47^. The total MMSE score ranges from 0 to 30 and can be performed within 10 min. According to the MMSE score, individuals were classified into good ($\ge 24$), moderate (11–23), and poor (0–10) cognitive capacity. MMSE score was used to exclude individuals whose score was ≤ 10. The LOTCA comprised of seven major sections (including orientation, visual perception, spatial perception, motor praxis, vasomotor organization, thinking operations, and attention and concentration) divided into 26 subsections, and each subsection was scored on a 4-point Likert scale. A combined score of each major section was computed by adding the raw scores of each related subsection. Finally, a total LOTCA score was computed by adding the total scores of all subsections. The total LOTCA score ranged between 27 and 123. A higher score represents better cognitive function. The LOTCA evaluation was carried out in accordance with the instruction manual^48^. Previously, the test was evaluated and validated in a variety of populations, including both Western^49^ and Arab populations^50^. The participants were divided into three groups based on their test scores: good (93–123), moderate (62–92), and poor (31–61).

### Blood analysis

Fasting blood samples were taken from all participants in the morning between 8 and 10 a.m., and were used to evaluate biomarkers (tHcy, α-tocopherol, γ-tocopherol, and oxidative stress markers) as detailed in the following subsections.

### Assessment of total homocysteine level (tHcy)

Plasma separated from fasting blood samples of the study populations was utilized to estimate the level of tHcy using high performance liquid chromatography (HPLC) as previously stated^51^. Normal values for adults’ range between 5.0 and 15.0 μmol/ l. A higher level of tHcy indicates poor health condition.

### Assessment of vitamin E level

Vitamin E was estimated as α- and γ-tocopherol in fasting serum samples of the participants using high-performance liquid chromatography paired with a diode array detector (Hitachi L-2455; Hitachi Ltd., Tokyo, Japan) (HPLC-DAD) with the help of α-tocopherol and γ-tocopherol standard (Sigma-Aldrich, Inc., St. Louis, MO, USA) as previously reported in literature^52^. The inter-assay coefficients of variation were 10.5% and 11.7% for serum α-tocopherol and γ-tocopherol, respectively. A low level of vitamin E indicates poor health condition.

### Assessment of oxidative stress parameters

#### Measurement of nitric oxide (NO)

Serum nitric oxide (NO) levels were measured as mentioned previously^53,54^. It was measured as nitrate and nitrite residues using Griess reagent, which gives a purple azo-compound when reacted with nitrite. The produced azo-compound was measured at 450 nm against a standard of sodium nitrate, and the results were stated in mmol/l. A low NO level may suggest a lack of physical exercise.

#### Measurement of total antioxidant capacity (TAC)

Serum total antioxidant capacity (TAC) was measured using Colorimetric Assay Kit (Catalog #K274-100; BioVision Incorporated; CA 95,035 USA). The antioxidant equivalent concentrations were measured at 570 nm as a function of Trolox concentration according to the manufacturer’s instructions.

$${\text{Sa/Sv}} = {\text{nmol/}}\mu {\text{l}}\,{\text{or}}\,{\text{mM}}\,{\text{Trolox}}\,{\text{equivalent}}$$

where: Sa is the sample amount (in nmol) read from the standard curve and Sv is the undiluted sample volume added to the wells. A higher value of TAC suggests a better health condition.

### Statistical analysis

The obtained data were statistically analyzed using SPSS 22.0 for Windows (SPSS Inc., Chicago, IL, USA), and and are presented as mean and standard deviation (SD) or standard error (SE). The multivariate analysis of variance was used to compare the testing variables. Between subjects’ effect of independent variables (i.e., physical activity levels and cognitive capacity) on biomarkers (e.g., serum -tocopherol and -tocopherol, TAC, NO, and plasma total homocysteine level) were investigated using analysis of covariance (ANCOVA), with age, BMI, gender, and educational status considered as covariates. The Bonferroni correction was used to rectify multiple comparisons. P-values less than 0.05 were considered statistically significant.

## Results

One hundred and six older adults aged 56–81 years were participated. Based on the level of PA, participants were divided into good, moderate, and poor activity levels. The descriptive statistics of the participants characteristics are shown in Table 1. Participants with low activity levels accounted for around 27% of the total. While almost 21% claimed having completed less than a high school education, more than 80% had finished a high school or university qualification. Participants with low activity levels showed a substantially higher BMI and waist-to-hip ratio (WHR) compared to other physically active groups ($p < 0.05$). The results showed that those with high and moderate activity levels had significantly lower glycosylated hemoglobin levels and higher maximum oxygen consumption (VO_2_ max) as a measure of physical fitness than those with low activity levels ($p < 0.05$). Additionally, persons with high or moderate PA levels showed significantly higher LTPA scores than those with low PA levels ($P < 0.05$). The LOTCA and MMSE scores of study participants were computed in order to assess cognitive capacity (Table 1). Individuals with high or moderate PA levels exhibited significantly higher total LOTCA and MMSE scores than those with low PA levels. Furthermore, people with high or moderate PA levels had significantly higher LOTCA scores in the five major sections (i.e., orientation, visual perception, spatial perception, vasomotor organization, and thinking operations) than those with low PA levels. People with high PA levels had significantly higher total LOTCA and MMSE scores, as well as all components of LOTCA scores except the attention and concentration score ($P < 0.05$).

**Table 1:** Participants characteristics based on the physical activity levels in older adults ($N = 106$).

Parameters	All samples	High^a^ (≥ 1200 METs-min/week)	Moderate^a^ (600–1199 METs-min/week)	Poor (< 600 METs-min/week)	ANOVA
N (%)	106 (100)	40 (37.7)	37 (34.9)	29 (27.4)	*p*
Educational level					
University	49	20	25	4	
High school	35	15	10	10	
Less than high school	22	5	2	15	
Male/Female sex	62/44	25/15	17/20	20/9	0.14
Age (years)	64.3 ± 7.5	64.9 ± 7.6	64.9 ± 7.9	62.7 ± 6.9	0.412
BMI (kg/m^2^)	23.2 ± 1.5	22.4 ± 1.4*^,†^	23.1 ± 1.3*	24.3 ± 1.3	0
Waist (cm)	82.1 ± 11.7	68.3 ± 11.3*	79.2 ± 11.6	98.8 ± 12.3	0.001
Hips (cm)	86.8 ± 11.9	81.5 ± 12.4*	87.3 ± 14.5	91.5 ± 9.1	0.003
WHR	0.94 ± 0.8	0.83 ± 0.7*	0.91 ± 0.8	1.1 ± 0.9	0.001
Systolic BP (mmHg)	108 ± 9.7	106.4 ± 12.7	102.1 ± 5.2	115.5 ± 11.1	0.238
Diastolic BP (mmHg)	74.3 ± 6.4	72.4 ± 6.7	71.7 ± 7.4	78.8 ± 5.2	0.212
VO_2_ max (ml/kg*min)	37.7 ± 6.4	41.1 ± 4.3*	39.6 ± 4.5*	30.7 ± 5.5	0
HbA1c (%)	3.6 ± 0.7	3.3 ± 0.6*	3.5 ± 0.6*	4.0 ± 0.8	0
LTPA (MET-H /week)	106.5 ± 5.7	145.5 ± 9.1*^,†^	115.2 ± 3.4*	58.7 ± 4.7	0
Biomarkers					
tHcy (μmol/l)	6.7 ± 2.6	4.3 ± 0.8*^,†^	7.4 ± 1.8*	9.3 ± 2.3	0
α-tocopherol (mg/L)	7.1 ± 2.6	9.5 ± 2.4*^,†^	6.2 ± 1.3*	4.9 ± 0.9	0
γ-tocopherol (mg/L)	1.1 ± 0.4	1.2 ± 0.4*^,†^	1.0 ± 0.3	0.9 ± 0.4	0
TAC (nmol /μL)	13.3 ± 6.5	20.6 ± 3.5*^,†^	10.1 ± 3.0*	7.3 ± 1.8	0
NO (µmol/L)	5.7 ± 1.3	6.7 ± 1.1*^,†^	5.4 ± 0.2	4.7 ± 0.9	0
Cognitive capacity					
Orientation	13.9 ± 2.2	15.6 ± 1.6*^,†^	13.7 ± 1.6*	11.8 ± 1.5	0
Visual perception	14.0 ± 2.7	16.2 ± 1.7*^,†^	13.5 ± 1.9*	11.7 ± 2.5	0
Spatial perception	13.1 ± 2.7	14.9 ± 1.6*^,†^	12.5 ± 2.4*	10.9 ± 2.7	0
Motor praxis	11.7 ± 2.9	13.4 ± 2.5*^,†^	11.1 ± 2.6	9.9 ± 2.6	0
Vasomotor organization	13.1 ± 2.6	14.9 ± 2.1*^,†^	12.7 ± 2.1*	11.1 ± 2.5	0
Thinking operations	14.4 ± 4.1	16.7 ± 3.5*^,†^	14.2 ± 3.2*	11.3 ± 3.4	0
Attention and concentration	2.6 ± 0.5	2.7 ± 0.5*	2.7 ± 0.5*	2.3 ± 0.4	0.002
Total LOTCA score	82.8 ± 13.7	94.9 ± 6.4*^,†^	80.5 ± 8.9*	69.1 ± 11.3	0
MMSE score	20.3 ± 5.7	25.1 ± 1.1*^,†^	19.6 ± 4.5*	14.7 ± 4.9	0

Values are expressed as a mean ± standard deviation (unadjusted scores).

BMI, body mass index; WHR, waist-to-hip ratio; BP, blood pressure; HbA1c, glycated hemoglobin; VO_2_ max, maximal oxygen consumption; METs, metabolic equivalents; LTPA, Leisure time physical activity; TAC, total antioxidant capacity (nmol/μL); tHcy, total homocysteine levels (μmol/L); NO, nitric oxide; MMSE, Mini-Mental State Examination; LOTCA, Loewenstein Occupational Therapy Cognitive Assessment.

*Significant difference between high or moderate and poor activity levels (*p* < 0.05).

^†^Significant difference between high and moderate activity levels (*p* < 0.05).

^a^Adjustment for multiple comparisons (Bonferroni).

People with high PA levels exhibited considerably greater blood levels of Vitamin E (α-tocopherol and γ-tocopherol), oxidative stress indicators (TAC and NO), and a considerably lower level of plasma tHcy compared to those with moderate or poor PA levels ($P < 0.05$). People with moderate PA levels had considerably higher TAC and lower plasma tHcy levels than those with low PA levels (Table 2).

**Table 2:** Associations between biomarkers (total homocysteine, α-tocopherol, γ-tocopherol, and oxidative stress markers) and physical activity levels in older adults (n = 106).

Variables	Physical activity levels^‡^			
	^b^High	^b^Moderate	Poor	*p*^a^
N (%)	40 (37.7)	37 (34.9)	29 (27.4)	
tHcy (μmol/l)	4.5 ± 0.3*^,†^	7.3 ± 0.3*	9.1 ± 0.3	0.000
α-tocopherol (mg/L)	9.1 ± 0.3*^,†^	6.3 ± 0.3*	5.1 ± 0.3	0.000
γ-tocopherol (mg/L)	1.2 ± 0.1*	1.0 ± 0.1	0.9 ± 0.1	0.010
TAC (nmol /μL)	20.4 ± 0.5*^,†^	9.8 ± 0.5*	7.9 ± 0.6	0.000
NO (µmol/L)	6.6 ± 0.2*^,†^	5.4 ± 0.2	4.8 ± 0.2	0.000

Values are expressed as mean ± SE (adjusted scores).

TAC, total antioxidant capacity (nmol/μL); tHcy, total homocysteine levels (μmol/L); NO, nitric oxide.

*Significant difference between high or moderate and poor activity levels.

^†^Significant difference between high and moderate activity levels

^‡^Age, sex, Educational level, and sex were used as a covariate.

^a^Analysis of covariance (ANCOVA).

^b^Adjustment for multiple comparisons (Bonferroni).

The current study's participants were also separated into three groups (good, moderate, and poor) based on their cognitive capacity as determined by LOTCA scores. People with high or moderate cognitive capacity had considerably greater blood levels of α-tocopherol, γ-tocopherol, and oxidative stress indicators (TAC and NO), as well as a significantly lower level of plasma tHcy than those with poor cognitive capacity ($P < 0.05$) (Table 3). People with high cognitive capacity had considerably greater serum levels of TAC than those with moderate cognitive capacity.

**Table 3:** Associations between biomarkers (total homocysteine, α-tocopherol, γ-tocopherol, and oxidative stress markers) and cognitive capacity in older adults (n = 106).

Variables	Cognitive capacity^‡^ (LOTCA scores)			
	^b^Good	^b^Moderate	Poor	*p*^a^
N (%)	28 (26.4)	62 (58.5)	16 (15.1)	
tHcy (μmol/l)	4.9 ± 0.3*^,†^	6.7 ± 0.2*	10.3 ± 0.5	0.000
α-tocopherol (mg/L)	8.3 ± 0.4*^,†^	6.8 ± 0.3	5.6 ± 0.6	0.354
γ-tocopherol (mg/L)	1.2 ± 0.1*	1.0 ± 0.1	0.8 ± 0.1	0.103
TAC (nmol /μL)	19.3 ± 0.9*^,†^	11.6 ± 0.6	8.8 ± 1.3	0.000
NO (µmol/L)	6.3 ± 0.2*^,†^	5.6 ± 0.1*	4.5 ± 0.3	0.060

Values are expressed as mean ± SE (adjusted scores).

TAC, total antioxidant capacity (nmol/μL); tHcy, total homocysteine levels (μmol/L); NO, nitric oxide; LOTCA, Loewenstein Occupational Therapy Cognitive Assessment.

*Significant difference between Good or Moderate and Poor cognitive capacity.

^†^Significant difference between Good and Moderate cognitive capacity.

^‡^Age, sex, educational level, and sex were used as a covariate

^a^Analysis of covariance (ANCOVA).

^b^Adjustment for multiple comparisons (Bonferroni).

The ANCOVA was used to evaluate between subjects’ effects of independent variables (i.e., PA levels and cognitive capacity) on biomarkers (i.e., α-tocopherol, γ-tocopherol, oxidative stress markers and tHcy). PA levels (high versus moderate versus poor) were associated with all biomarkers (all *p* = 0.000, and *p* = 0.010 for γ-tocopherol) (Table 2). While tHcy and TAC were significantly correlated with individuals’ cognitive capacity, vitamin E levels and NO were not related to cognitive capacity in older adults (Table 3). There was no interaction between PA levels and cognitive capacity on the effects of biomarkers. There were no significant effects of age, sex, BMI, and educational status on all biomarkers (*p* > 0.05).

## Discussion

In the present study, the participants with poor PA levels had higher BMI and WHR compared to high and moderate PA levels. Similarly, Han et al.^55^ found that individuals who were less physically active had higher BMI and WHR. The association between high WHR and physical inactivity is thought to be related to increased abdominal fat deposition and skeletal muscle atrophy in physically inactive subjects^55^. Participants with high and moderate PA levels exhibited lower glycosylated hemoglobin levels and higher VO_2_ max than those with low PA levels. Previous research found a significant association between PA and aerobic power in both young and older people^56–58^. Additionally, Young et al.^59^ found that physically active people had lower random and fasting glucose and glycated hemoglobin levels than physically inactive persons^59^. In the current study, 15% of the participants exhibited poor cognitive capacity. Almost 60% of participants had moderate cognitive capacity, while others had good cognitive capacity. A prior study found that 25–50 percent of older persons had poor cognitive capacity, and that older age was associated with poor cognitive capacity^60^.

In this study, individuals with poor cognitive capacity had considerably lower amounts of vitamin E (α-tocopherol and γ-tocopherol) than participants with moderate or good cognitive capacity. A decreased amount of vitamin E (α-tocopherol and γ-tocopherol) did not, however, have a significant relationship with cognitive capacity in older persons. Previous studies have found low levels of Vitamin E in elderly people with Alzheimer’s disease and mild cognitive impairment^61,62^. Another study discovered a relationship between increased vitamin E levels and a reduced incidence of cognitive impairment in the elderly^52^. Their findings imply that increased levels of vitamin E (especially, α-tocopherol and γ-tocopherol) may play a preventive role in lowering the risk of cognitive impairment^52^. Interestingly, an Italian study found that older persons with greater levels of vitamin E (e.g., γ-tocopherol) had a decreased risk of cognitive impairment^63^. Furthermore, Bowman et al.^64^ studied older persons and discovered that biomarker patterns in plasma were related to brain volume and cognitive function. Moreover, they discovered that a high level of vitamin E (e.g., α-tocopherol) in plasma was associated with less brain shrinkage and greater cognitive function^64^.

This study reveals significantly lower levels of TAC in people with moderate and poor cognitive capacity compared to good cognitive capacity. Additionally, a lower level of TAC was significantly associated with cognitive capacity in older adults. A recent study indicates that a lower level of TAC was associated with poor cognitive capacity in older adults^9^. Few studies examined the association between TAC and cognitive impairment in older adults. For instance, one study has suggested that lower level of TAC was independently correlated with less grey matter in the middle temporal lobe in patients with mild cognitive impairment^65^. Another study supported this finding, revealing a considerably lower brain metabolic rate in the inferior and temporal lobes of patients with weak cognitive function when compared to a matched healthy group^66^.

The current investigation discovered that those with poor cognitive capacity had considerably greater levels of plasma tHcy than persons with moderate or good cognitive capacity. Furthermore, higher levels of tHcy in plasma were linked to poor cognitive performance in older persons. Previous research suggests that hyperhomocysteine may impair cognitive function in older persons^67,68^. Another study examined the relationship between tHcy levels and cognition in older adults, and found an association between hyper-homocysteine and poor cognition in people aged 60 and above^69^. Similarly, a normative aging study in older adults (aged 54–81 years) revealed that persons with hyper-homocysteine levels performed poorly on spatial copying skills^70^. Furthermore, hyperhomocysteine levels were found to be substantially associated to poor performance on a variety of cognitive tests in older persons^71^. Although a previous review found hyper-homocysteine to be an independent risk factor for cognitive dysfunction, vascular dementia, and Alzheimer’s disease, there was no evidence of a causative association between homocysteine levels and cognitive impairments^72^. However, a variety of biological mechanisms have been identified that may connect elevated tHcy to cognitive impairment^19^, including vascular mechanisms, regional brain atrophy, the development of neurofibrillary tangles and amyloid plaques, neuronal death, and epigenetic mechanisms, among others^20,73,74^. The mechanisms are not necessarily exclusive, and many distinct pathways are likely to be implicated, as explained in detail in the published review^20^. Since elevated tHcy has long been considered a risk factor for cardiovascular disease^75^, it was logical that early studies attributed the connection between tHcy and dementia to damage to the cerebral vasculature rather than Alzheimer’s disease pathology^76^. Additionally, according to another hypothesis, increased tHcy contributes to the inhibition of methylation reactions in which S-adenosylmethionine is the methyl donor^77^. Given the vast number of methylation reactions in the body, this mechanism could have many further impact^20^. Depending on the relative affinities of the substrate and S-adenosylhomocysteine for a specific methylase, some methylation reactions are likely to be more susceptible to S-adenosylhomocysteine inhibition than others^20^. In short, there are several causal pathways that is supported by the vast number of possible mechanisms by which homocysteine could damage the brain. This could be one of the reasons why high tHcy is such a major risk factor for dementia^20^.

The current study suggests that individuals with good PA levels had higher serum levels of α-tocopherol, γ-tocopherol, and oxidative stress markers (TAC and NO), as well as a lower level of plasma tHcy than those with moderate or poor PA levels. A previous study found that consistent PA has a favorable effect on the antioxidant system and may reduce lipid peroxidation^78^. Another study found that 24 weeks of moderate aerobic exercise enhanced antioxidant capacity and reduced oxidative stress free radicals^79^.

There were significant associations between PA levels and cognitive capacity in older adults. Participants with good or moderate PA levels showed significantly higher LOTCA and MMSE scores than those with poor PA levels. Gill et al.^80^ discovered a link between higher PA levels and better cognitive function in middle-aged and older persons^80^. A longitudinal study found an association between poor cardiorespiratory fitness at baseline and lower cognitive function 6 years later^81^. Another study found a lower incidence of cognitive impairment in people who exercised at least three times a week at a higher intensity than walking over a 5-year follow-up period^82^. Abbott et al.^83^ reported a 1.8 times increased risk of developing dementia in older people who walked $< 0.25~\,{\text{miles/day}}$ compared to people walking $~ > 2\,{\text{miles/day}}$. In addition, Larson and colleagues reported a reduced risk of developing dementia or Alzheimer’s disease in older people who exercised three or more times per week^84^. Furthermore, Bielak et al.^85^ found a link between baseline cognition and physical activity: persons who exercised more had higher baseline cognitive scores than those who did not.

The current study has a few limitations. First, because it was a cross-sectional study, no conclusions can be drawn about the causal association between vitamin E, oxidative stress indicators, tHcy levels, or PA and cognitive function. Second, the level of PA was not measured objectively, such as with an accelerometer. As a result, it is unknown whether all people completed the same level of PA. A prior study found that the intensity of PA has a significant impact on cognitive function in older persons^86^. Future studies may employ a direct measure of PA, such as an accelerometer, or any other sensor (e.g., mobile heart rate monitors), to evaluate the influence of these biomarkers on cognitive deterioration in older persons with varying amounts of PA.

## Conclusion

This study concludes that physical activity level was associated with Vitamin E, oxidative stress markers, total homocysteine levels, and cognitive capacity in older persons. Although cognitive capacity was associated with total homocysteine and total antioxidant capacity, it was unrelated to vitamin E levels and nitric oxide in older persons. These biomarkers are the most reliable indicators of cognitive function, and their measurement can provide a more accurate assessment of cognitive function in older persons. However, more research is needed to determine the link between high plasma homocysteine levels and poor cognition or physical activity-related outcomes. If future research confirms our findings, proof of a causal link between plasma homocysteine and cognitive capacity development would require further elucidation of pathophysiologic mechanisms. It would also be intriguing to see concrete evidence from human controlled clinical trials that therapies to lower plasma homocysteine levels can minimize the likelihood of cognitive deterioration in older persons.

## Data Availability

All data generated or analyzed during this study are presented in the manuscript. Please contact the corresponding author for access to data presented in this study.
